# Familial Mediterranean Fever: A Diagnostic and Therapeutic Challenge

**DOI:** 10.7759/cureus.98777

**Published:** 2025-12-08

**Authors:** Robin Sia

**Affiliations:** 1 Rheumatology, Northern Hospital, Epping, AUS

**Keywords:** autoinflammatory disease, familial mediterranean fever, fmf, periodic fever syndrome, recurrent pericarditis, serositis

## Abstract

Recurrent pericarditis is a complex and often debilitating clinical entity characterized by repeated episodes of pericardial inflammation following an initial acute insult. The condition poses a significant diagnostic and therapeutic challenge, as its underlying causes span a wide spectrum of etiologies, including autoimmune disorders (such as systemic lupus erythematosus and rheumatoid arthritis), infectious processes (notably viral, bacterial, or tuberculous), neoplastic involvement, and a growing recognition of autoinflammatory mechanisms. While the majority of recurrent pericarditis cases are deemed idiopathic, presumed to reflect a post-viral or immune-mediated phenomenon, there is increasing evidence that a subset of patients may have underlying monogenic autoinflammatory syndromes. We present the case of a middle-aged woman with recurrent, steroid-responsive pericarditis accompanied by episodic fever and elevated inflammatory markers, in whom conventional infectious and autoimmune causes were excluded. Her clinical course raised suspicion of an underlying autoinflammatory mechanism, highlighting the importance of considering familial Mediterranean fever (FMF) and other hereditary periodic fever syndromes in patients with otherwise unexplained, relapsing pericardial disease.

## Introduction

Recurrent pericarditis represents a challenging clinical entity characterised by repeated episodes of pericardial inflammation following an initial event, often leading to significant morbidity and prolonged corticosteroid exposure. The condition has a broad differential diagnosis, encompassing autoimmune, infectious, neoplastic, and autoinflammatory causes. While most cases are classified as idiopathic, presumed viral, or immune-mediated, there is increasing recognition of an underlying autoinflammatory component in a subset of patients previously considered idiopathic. In particular, hereditary periodic fever syndromes such as familial Mediterranean fever (FMF), tumor necrosis factor receptor-associated periodic syndrome (TRAPS), and mevalonate kinase deficiency (MKD) have been implicated in recurrent pericardial inflammation, often in the absence of overt serositis elsewhere. We report the case of a middle-aged woman with steroid-responsive recurrent pericarditis associated with recurrent fevers and elevated inflammatory markers, in whom a conventional infectious, neoplastic, and autoimmune work-up was unrevealing. However, eventually a probable diagnosis of FMF was made following genetic testing. This case underscores the importance of recognizing autoinflammatory causes of pericarditis, as diagnosis has critical therapeutic implications, with targeted therapies such as colchicine and interleukin-1 blockade (e.g., anakinra, rilonacept) offering potential for long-term remission and steroid-sparing benefit.

## Case presentation

**Figure 1:**
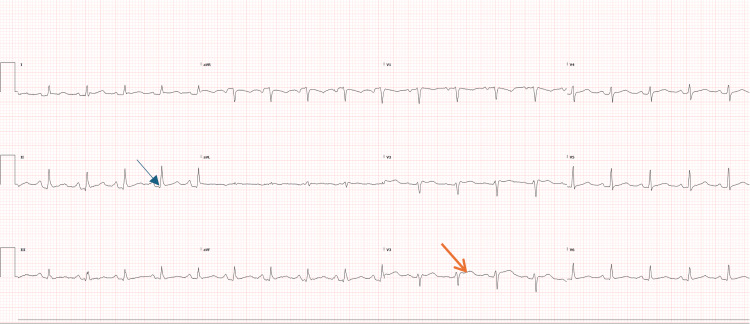
ECG showing diffuse ST-elevation (orange arrow) and PR-depression (blue arrow) in keeping with acute pericarditis.

**Figure 2:**
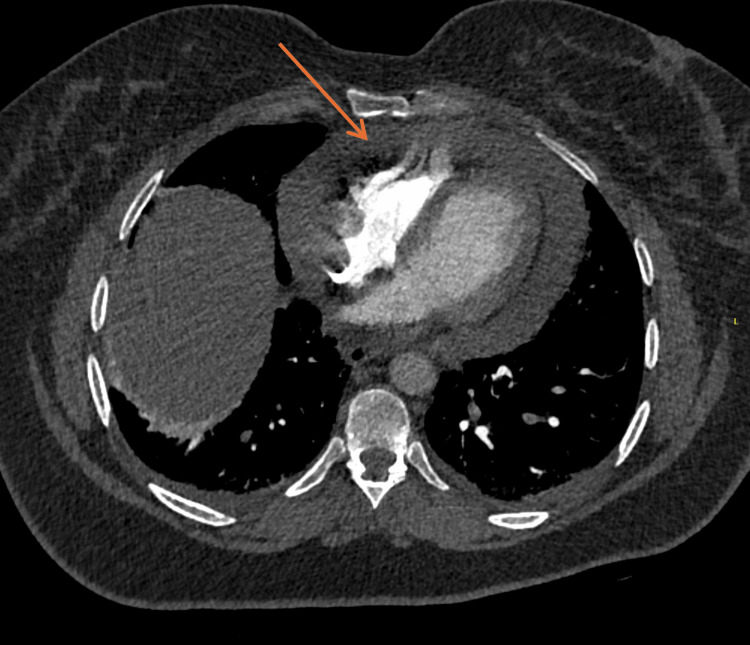
CT-pulmonary angiography demonstrating large pericardial effusion (orange arrow).

A 55-year-old woman with a history of hypothyroidism on levothyroxine presented with recurrent episodes of sharp, pleuritic chest pain in keeping with acute pericarditis associated with intermittent fever and hypotension. She was first admitted in July 2025 with acute pericarditis, where her electrocardiogram (ECG) showed diffuse ST-elevation and PR-depression (Figure 1), complicated by large pericardial effusion with potential tamponade physiology on CT-pulmonary angiography (CTPA) (Figure 2), requiring a left mini-thoracotomy and pericardial window procedure that drained 400 mL of serous fluid. Pericardial fluid analysis revealed reactive mesothelial cells, histiocytes, and dense mixed inflammation with numerous neutrophils but no malignant cells or granulomas. Pericardial and tissue cultures showed no growth, and tuberculosis polymerase chain reaction (PCR) was negative. Histopathology demonstrated fibroadipose tissue with fibrin and neutrophilic inflammation, mild chronic inflammatory infiltrates composed of histiocytes and lymphocytes, and rare eosinophils, without necrosis or granuloma formation. She was treated empirically with intravenous benzylpenicillin and flucloxacillin for two days before being discharged on 0.5 mg twice a day colchicine for three months and ibuprofen 400 mg thrice a day for two weeks.

Within two weeks, she was readmitted twice for recurrent pericarditis. On her second admission in early August 2025, she presented with severe, sharp chest pain and hypotension (70/40 mmHg). CTPA demonstrated a moderate pericardial effusion with small bilateral pleural effusions and no pulmonary embolism. Bedside ultrasound confirmed a 1.2 cm circumferential effusion without significant re-accumulation. Transthoracic echocardiography revealed a moderate circumferential effusion (up to 1.5 cm posteriorly) with mild hemodynamic changes, late diastolic right-ventricular collapse, and a normal inferior vena cava. Blood and pericardial fluid cultures remained negative. Given the recurrence, she was commenced on high-dose prednisolone (50 mg daily), colchicine 0.5 mg twice daily, and a proton-pump inhibitor. Doxycycline was added empirically for possible *Coxiella burnetii *infection while awaiting repeat Q-fever serology, which ultimately returned negative.

Her past history was otherwise unremarkable, and she denied any features suggestive of connective-tissue disease. There was no family history of autoinflammatory conditions such as FMF. She had not recently experienced upper respiratory tract infections or gastrointestinal symptoms and had tolerated colchicine without adverse effects. She recalled that symptoms began approximately one week into an overseas pilgrimage, suggesting a possible infectious trigger.

Laboratory results during her third admission showed persistently elevated inflammatory markers, with an ESR of 92 mm/h (normal: <21 mm/h) and CRP of 184 mg/L (normal: <6 mg/L). Her renal function was normal, and liver tests demonstrated mild cholestatic changes with a normal bilirubin. Her full blood count showed neutrophilia and reactive thrombocytosis. Autoimmune screening revealed an ANA titer of 1:320 (homogeneous pattern), with negative extractable nuclear antigens (Ro/SSA, La/SSB, Sm, RNP, Jo-1, and Scl-70), rheumatoid factor, and anti-cyclic citrullinated peptide antibodies. dsDNA was indeterminate, and complement levels were normal. ANCA, HIV, and extended viral PCRs were negative. Repeat Q-fever serology was negative for *Coxiella burnetii*, as were hepatitis B, hepatitis C, and tuberculosis PCR. Thyroid function tests were normal. Repeat echocardiography on August 6 showed preserved left-ventricular systolic function and only a small residual pericardial effusion without tamponade physiology.

The differential diagnosis included idiopathic or post-viral pericarditis, autoimmune pericarditis, and autoinflammatory causes such as FMF. Although she had a positive ANA, there were no other features of systemic autoimmune disease despite the indeterminate anti-dsDNA and positive ANA, including Raynaud's phenomenon, alopecia, peripheral synovitis, oral ulcers, sicca symptoms, or photosensitive rash. Given the pattern of recurrent, steroid-responsive pericarditis with fever, an autoinflammatory mechanism was considered, and MEFV gene testing was arranged. Infectious causes, including Q fever and tuberculosis, were effectively excluded.

She was managed with a gradual taper of prednisolone (50 mg for two weeks, followed by 37.5 mg, 25 mg, and 12.5 mg at two-week intervals) while continuing colchicine and gastric protection with a proton pump inhibitor. Trimethoprim-sulfamethoxazole was initiated for pneumocystis prophylaxis, with plans to discontinue it once her corticosteroid dose fell below 20 mg daily. She was reviewed regularly in the cardiology, rheumatology, and infectious diseases clinics, with serial CRP measurements and echocardiographic monitoring. During the prednisolone taper, she experienced a flare requiring pulse methylprednisolone 250 mg daily for three days, underscoring the challenge of achieving stable disease control in FMF. She remains on a slower corticosteroid wean, with her colchicine increased to 0.5 mg three times daily, and plans to commence conventional synthetic disease-modifying antirheumatic drugs (csDMARDs) such as azathioprine, methotrexate, or mycophenolate mofetil to support long-term disease control.

Recurrent pericarditis is often idiopathic, but in some cases represents an autoinflammatory process rather than classic autoimmune or infectious inflammation. Familial Mediterranean Fever, caused by mutations in the MEFV gene, can present with recurrent sterile serositis such as peritonitis, pleuritis, or pericarditis. Although classically seen in individuals of Mediterranean descent, FMF can occur in other populations. The prompt steroid responsiveness, recurrent febrile nature, and lack of alternative etiology in this patient warrant evaluation for an underlying autoinflammatory disorder. Furthermore, the patient meets the Tel Hashomer criteria for FMF, which include a combination of major features such as recurrent febrile episodes with serositis (peritonitis, pleuritis, or monoarthritis) and minor features including supportive symptoms like erysipelas-like erythema or a favorable response to colchicine; together, these clinical criteria establish a high-specificity diagnosis of FMF. On further testing, a pathogenic mutation was identified in the MEFV gene (which is associated with FMF). Specifically, there is a single base change at position 2082 in the coding sequence, from guanine (G) to adenine (A). This results in the substitution of the amino acid methionine (Met) with isoleucine (Ile) at position 694 of the protein (p.Met694Ile).

This case illustrates the diagnostic complexity of recurrent pericarditis and emphasizes the importance of considering autoinflammatory causes such as FMF, particularly when conventional investigations are unrevealing. Early recognition allows for targeted therapy with colchicine and, if necessary, interleukin-1 inhibitors to prevent further recurrences or constrictive complications.

## Discussion

FMF is the most common hereditary autoinflammatory disorder, characterised by recurrent, self-limited attacks of fever and inflammation involving the peritoneum, pleura, joints, or skin. It primarily affects individuals of Mediterranean and Middle Eastern ancestry, particularly Turkish, Armenian, Arab, and Sephardic Jewish, but has also been increasingly recognized in other populations due to migration and better genetic screening [1]. The disease usually begins in childhood, with about 90% of cases manifesting before the age of 20, although late-onset forms have been described. Attacks typically last 12-72 hours and resolve spontaneously, with symptom-free intervals between episodes [1].

FMF arises from mutations in the MEFV gene located on chromosome 16p13.3, which encodes pyrin (also called marenostrin), a regulatory protein in the innate immune system. Pyrin controls the activation of the inflammasome and subsequent interleukin-1β release. Gain-of-function MEFV mutations lead to inappropriate inflammasome activation, excessive interleukin-1β production, and recurrent inflammatory flares. Although classically inherited in an autosomal recessive manner, heterozygotes may display symptoms, suggesting variable penetrance and modifier influences such as epigenetic factors [1-3]. The most severe mutation, M694V, is strongly associated with early disease onset, colchicine resistance, and increased risk of developing AA amyloidosis, the most feared long-term complication of FMF [1,3]. The mutation M694I is also recognized as pathogenic and can produce a classic FMF phenotype, as in this case.

Clinically, FMF presents with abrupt episodes of fever accompanied by severe abdominal pain due to peritonitis, unilateral pleuritic chest pain from pleuritis, monoarthritis (often of the lower limbs), and occasionally an erysipelas-like rash over the legs. Between attacks, patients may appear completely well, although subclinical inflammation, manifested by persistently elevated CRP or serum amyloid A (SAA), may persist [1,4]. Common triggers for FMF flares include stress, infections, strenuous exercise, menstruation, cold exposure, and non-adherence to colchicine, all of which can precipitate inflammatory attacks in genetically susceptible individuals. The most serious long-term complication is secondary AA amyloidosis, primarily affecting the kidneys and leading to proteinuria and renal failure if left untreated. Other reported complications include pericarditis and increased cardiovascular risk due to chronic systemic inflammation [1,3,4].

Diagnosis is primarily clinical, guided by recurrent febrile serositis episodes and supported by genetic testing for MEFV mutations. The Tel-Hashomer criteria, along with the EuroFEVER (European Registry of Autoinflammatory Diseases) and PRINTO (Paediatric Rheumatology International Trials Organisation) criteria, are among the most widely used diagnostic frameworks [1,4]. Laboratory findings during attacks typically include neutrophilia and elevated ESR, CRP, and SAA. Genetic confirmation can aid diagnosis and prognosis, but a negative MEFV result does not exclude FMF, as up to 10%-20% of clinically diagnosed patients may lack identifiable mutations [1,3]. The main differential diagnoses include other periodic fever syndromes such as TRAPS, cryopyrin-associated periodic syndromes (CAPS), and mevalonate kinase deficiency [2,4].

The cornerstone of FMF management is lifelong colchicine therapy, which dramatically reduces the frequency and severity of attacks and prevents amyloidosis [1,3]. Standard dosing is adjusted for age, renal function, and tolerability. Gastrointestinal side effects are common but manageable, and monitoring for myotoxicity is essential, especially when used concurrently with other drugs like statins. For patients who are colchicine-resistant or intolerant (5%-10% of cases), biologic therapies targeting IL-1β, such as anakinra, canakinumab, or rilonacept, have proven effective in reducing attacks and systemic inflammation [2,5]. The phase 3 CLUSTER (Canakinumab for the Treatment of Autoinflammatory Recurrent Fever Syndromes) trial demonstrated the safety and sustained efficacy of canakinumab in colchicine-resistant FMF, supporting its role as a second-line therapy [5].

Prognosis in FMF is excellent when colchicine is taken consistently, with markedly reduced mortality and amyloidosis rates [1,3]. Poor compliance remains the most significant risk factor for complications. The disease course is variable; some patients experience decreasing attack frequency over time, while others require escalation to biologic therapy [1,5,6]. Emerging research focuses on epigenetic regulation of MEFV, potential biomarkers of disease activity (such as SAA or interleukin-18 levels), and “treat-to-target” strategies that aim to suppress subclinical inflammation and prevent amyloidosis [2,6].

## Conclusions

In summary, FMF is a monogenic autoinflammatory disease driven by pyrin inflammasome dysregulation, resulting in recurrent febrile serositis and risk of secondary amyloidosis. It exemplifies the interface between genetics and innate immunity, and timely recognition, particularly in patients of Mediterranean ancestry, combined with early colchicine therapy, can transform prognosis. Ongoing research continues to refine the understanding of genotype-phenotype variability, optimize biologic therapies for resistant cases, and explore personalized approaches to disease monitoring and prevention.
